# Outlook of Adipose-Derived Stem Cells: Challenges to Their Clinical Application in Horses

**DOI:** 10.3390/vetsci10050348

**Published:** 2023-05-12

**Authors:** Valeria Petrova, Ekaterina Vachkova

**Affiliations:** Department of Pharmacology, Animal Physiology and Physiological Chemistry, Faculty of Veterinary Medicine, Trakia University, 6000 Stara Zagora, Bulgaria; valeria.petrova1075@gmail.com

**Keywords:** equine, species-specific stemness features, regenerative therapy

## Abstract

**Simple Summary:**

Athletic horses are often exposed to traumatic injuries, resulting in severe financial losses. Adipose tissue possesses a high potential as an easily accessible source and provides a higher yield of mesenchymal stem cells for various applications in regenerative medicine. Concerning the identification of the stemness features of isolated cells, some of the most commonly applied standards are not applicable because of the species-specific responses to the differentiation protocols. In many cases, the cells cannot reveal their multipotent properties, so their stemness features remain questionable. The adaptation, optimization, and standardization of equine-specific protocols for cell isolation and culture conditions are also discussed. The presented new approaches elucidate the possibility of the transition from cell-based to cell-free therapy with regenerative purposes in horses as an alternative treatment to cellular therapy. The current review summarizes aspects of the specificity of equine adipose stem cells concerning their features, immunophenotyping, secretome profile, differentiation abilities, culturing conditions, and consequent possibilities for clinical application in some equine-specific disorders.

**Abstract:**

Adipose tissue is recognized as the major endocrine organ, potentially acting as a source of mesenchymal stem cells for various applications in regenerative medicine. Athletic horses are often exposed to traumatic injuries, resulting in severe financial losses. The development of adipose-derived stem cells’ regenerative potential depends on many factors. The extraction of stem cells from subcutaneous adipose tissue is non-invasive, non-traumatic, cheaper, and safer than other sources. Since there is a lack of unique standards for identification, the isolated cells and applied differentiation protocols are often not species-specific; therefore, the cells cannot reveal their multipotent properties, so their stemness features remain questionable. The current review discusses some aspects of the specificity of equine adipose stem cells concerning their features, immunophenotyping, secretome profile, differentiation abilities, culturing conditions, and consequent possibilities for clinical application in concrete disorders. The presented new approaches elucidate the possibility of the transition from cell-based to cell-free therapy with regenerative purposes in horses as an alternative treatment to cellular therapy. In conclusion, their clinical benefits should not be underestimated due to the higher yield and the physiological properties of adipose-derived stem cells that facilitate the healing and tissue regeneration process and the ability to amplify the effects of traditional treatments. More profound studies are necessary to apply these innovative approaches when treating traumatic disorders in racing horses.

## 1. Introduction

**Figure 1:**
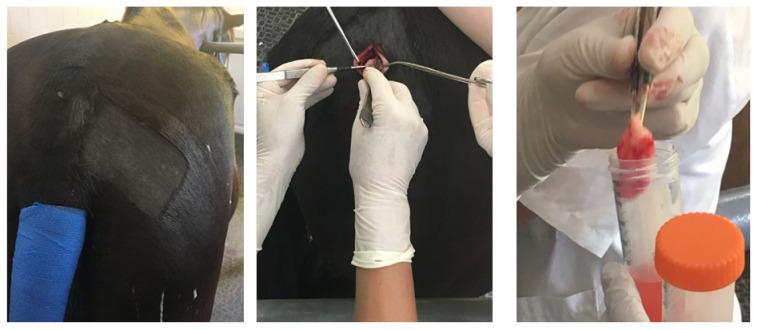
The main stages of surgical biopsy of subcutaneous fat tissue depot located in *Regio radicis caudae* in a horse.

Adipose tissue is an abundant and convenient source of mesenchymal stem cells (MSCs) [1] that, together with those derived from bone marrow (BMSCs), possess a higher potential for application in cell-based therapy, where the primary purpose is to provoke and support regenerative processes in damaged tissues [2,3]. To date, in veterinary medicine, stem cells from adipose tissue have successfully been used mainly in horses and dogs for treating tendons and joint injuries, bone defects, musculoskeletal disorders, and even some kidney and ophthalmic diseases [3,4,5,6]. Obtaining material for the isolation of MSCs from adipose tissue (Figure 1) is relatively non-invasive and non-traumatic; liposuction surgery is a cheaper and safer method than bone marrow aspiration [7].

The higher content of MSCs in adipose tissue compared to bone marrow in large animals, such as horses, explains its preferability as a source of stem cells, due to the abundant amount of cell mass required to achieve a therapeutic effect [8]. Furthermore, the amount of stem cells in one gram of subcutaneous adipose tissue is 500 times higher than what can be obtained from the same quantity of bone marrow aspirate [9]. For cell-based therapy in horses, autologous bone marrow mesenchymal stromal cells are used due to their differentiation into different cell types and their ability to reproduce regenerative processes at the site of damage in difficult-to-repair tissues such as tendons and ligaments [10]. The same abilities have been established for ASCs from lipoaspirates in humans when compared to bone marrow MSCs [11]. In the event that the amount of the isolated cells from lipoaspirates exceeds that from bone marrow, the adipose tissue would be the preferable source of MSCs.

***Stem cells’ classification and mesenchymal stem cells (MSCs) discovery***: In general, stem cells can be classified depending on their origin (embryonal, adult, and induced pluripotent stem cell, [12], or to their differentiation potential (totipotent, pluripotent, multipotent, oligopotent, and unipotent) [13]. The last one does not possess any ability for multilineage differentiation, except at the site of their origin. MSCs in particular are multipotent adult stem cells that can differentiate into various mesodermal cell types, including adipocytes, chondrocytes, and osteoblasts [14,15].

First identified in bone marrow, MSCs can also be obtained from almost all tissues and organs of the adult organism or the fetus: adipose tissue [16]; umbilical cord blood [17]; peripheral blood [18,19]; the dermis or dental pulp [20,21]; or skeletal muscle [22].

***Features of MSCs***: Following the discovery of MSCs, it was found that at low seeding densities, individual precursors can proliferate to generate new colonies of cell structures known as colony forming units-fibroblast (CFU-F), which are inherent in stem cells [23] and are considered as the gold standard in the analysis of their identification. In humans, for example, one cell could produce only a single colony, whereas in mice and rats, one cell may reproduce into multiple colonies [24]. However, in horses, even at a seeding density of 100 cells, colony formation is almost non-existent [25].

The knowledge of stem cell properties and features has improved over the years. Researchers still face the Gordian Knot in finding an algorithm for equalizing the quality and homogeneity of animal MSCs to increase their clinical efficacy. To address this problem, the International Society for Cellular Therapy (ISCT) has published minimal criteria, according to which MSCs must meet the following standards: the ability to attach to the surface of the vessel during cultivation; multipotent potential for differentiation, i.e., the cells must have the ability to differentiate into osteoblasts, chondrocytes and adipocytes (cells of mesodermal origin); expression of specific surface antigens (CD-cluster of differentiation, superficially located on the cell membrane glycoproteins), with more than 91% of the MSC population having to express markers for less the differentiated cells CD73, CD90, and CD105 and not expressing (be negative) markers specific for endothelial and hematopoietic cells CD34, CD45, CD14 or CD11b, CD19 or CD79α and HLA-DR [26].

***Immunophenotyping of ASCs***: Adipose-derived stromal and stem cells (ASCs) are mesenchymal stem cells (MSCs) that exhibit similar properties since they adhere to plastic culture flasks, can be expanded in vitro, and may differentiate into multiple cell lineages [27]. ASCs include different subgroups associated with their various functions, e.g., as precursors of adipocytes or vascular support cells. Therefore, it is difficult to reconcile a definite and independent expression profile [7]. Under the authority of the International Federation of Adipose Therapeutics (ISCT), international standards have been developed based on reproducible parameters as minimal definitions of stromal cells, wherein they are evaluated both as an uncultured stromal vascular fraction (SVF) and as an adherent stromal/stem cell population. The further expansion of this fraction gives rise to an adherent cell population termed adipose tissue-derived stromal cells (ASCs). Accordingly, to be accepted as ASCs, the cellular population should be negative (<2%) for hematopoietic markers such as CD11b and CD45 and positive (>90%) for stromal markers such as CD13, CD73, and CD90 [1]. The latter allows us to conclude that, as in humans, the aforementioned CD73, CD105, CD44, and CD90 can also be considered markers for identifying ASCs in horses. The CD29 marker should also be included because over 90% of the cells have been found positive, confirmed by flow cytometric and qPCR analysis [28].

As negative markers for equine ASCs have been identified, the hematopoietic marker CD45 is expressed by monocytes and macrophages, CD14, and endothelial marker CD31 [28]. In human ASCs, the hematopoietic marker CD34 has shown positive expression, which tends to decrease with the increasing number of passages [29]; in horses, however, such a trend has not been explored. Some authors propose additional markers that have not been previously studied, such as CD61, CD91, CD228, and CD315, in adipose and bone marrow MSCs in humans and horses [30].

In addition to the above-mentioned CD markers, stemness transcriptional factors such as NANOG, OCT4, SOX2, REX1, NOTCH1, and NESTIN should also be investigated at present [31]. To characterize human ASCs, new approaches such as flow cytometry, quantitative PCR, transcriptome sequencing [32], the evaluation of cell surface proteins by mass spectrometry [33,34], and the determination of ASCs’ secretome profile [35] have been used. In horses, the combination of enrichment of the MSCs surface proteome by biotinylation and consequent MS analysis has been reported as a valuable alternative to immunophenotyping surface markers when suitable antibodies are not available [30]. In other words, the requirements for ASCs increase, improving their efficacy in clinical application.

To date, the detailed expression profile of ASCs is still arguable [36] and very complicated, especially in animals, since it depends on various factors arising from the microenvironmental extracellular conditions, isolation methods, and tissue origin.

**Figure 2:**
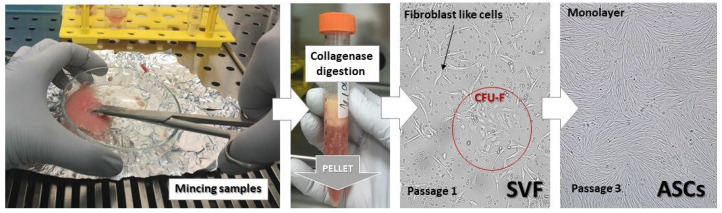
Stages of isolating procedure for equine adipose stem cells. **Abbreviations:** SVF—stromal vascular fraction; CFU-F—colony forming unit fibroblast; adipose stem cells (ASCs).

***Features of ASCs***: To a large extent, the characteristics of ASCs overlap with those common in mesenchymal stem cells due to their similar embryonic origin. However, they have some specific features that need to be addressed. An older method developed to isolate ASCs from white adipose tissue in humans is still in use, whereas in 2001, Zuk et al. identified those cells as MSCs [37,38]. Accordingly, adipose tissue is mechanically minced and then subjected to enzymatic digestion with collagenase, which disrupts the peptide bonds in the collagen molecules to release cells and centrifuge. The resulting pellet (Figure 2) is referred to as stromal vascular fraction (SVF), which contains stem cells, endothelial cells, endothelial progenitor cells, pericytes, smooth muscle cells, leukocytes, and erythrocytes [39].

There are multiple terms for stem cells derived from adipose tissue, for example preadipocytes, adipose-derived stromal cells, processed lipoaspirate cells, adipose-derived mesenchymal stem cells, and adipose-derived adult stem cells. The International Fat Applied Technology Society has adopted the term “adipose-derived stem cells” (ASCs) to identify the isolated-from-fat-tissue, plastic-adherent, multipotent cell population [40].

After seeding in culturing plates, the SVF cells give rise to a subset of elongated cells, which are less heterogeneous [1,41], adherent to plastic, easily cultivated and expanded in vitro, and whose average doubling time is approximately 2–5 days, depending on the number of passage and culturing conditions [29,42]. The ASCs can easily cryopreserve in a medium containing serum and dimethyl sulfoxide (DMSO) while retaining their proliferation and differentiation ability after defrosting [43].

As already mentioned, ASCs have a higher proliferative and adipogenic capacity than BMSCs, which are easier to differentiate into chondro- and osteogenic directions [44]. With appropriate inducers and under favorable microenvironmental conditions, ASCs can be differentiated even in cardiomyocytes [45,46].

**Figure 3:**
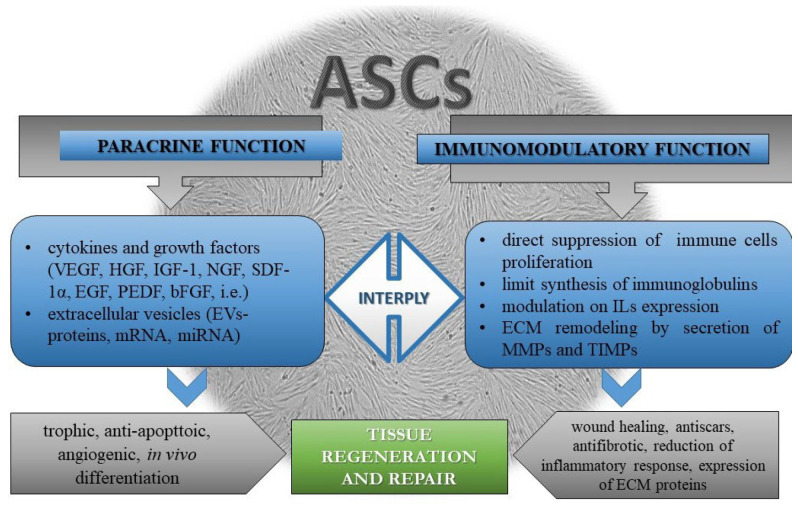
Schematic representation of the main mechanisms for tissue regeneration and repair in which ASCs are involved. **Abbreviations:** ASCs (adipose stem cells); bFGF (basic fibroblast growth factor); ECM (extracellular matrix); EVs (extracellular vesicles); HGF (hepatocyte growth factor); IGF-1 (insulin-like growth factor); ILs (interleukins); MMPs (matrix metalloproteinases); mRNA (messenger ribonucleic acid); miRNA (micro-ribonucleic acid); NGF (nerve growth factor); PDGF (platelet-derived growth factor); SDF -1α (stromal cell-derived factor); TIMPs (tissue inhibitors of metalloproteinases); VEGF (vascular endothelial growth factor).

Bioactive ASC products have clinical significance; the main healing effects of ASCs are due to their paracrine function and immunomodulation at the application site (Figure 3).

As part of the MSC community, ASCs also produce many cytokines, growth factors, and biologically active molecules, the spectrum of which largely overlaps with those of other MSCs. Many signals from the local microenvironment could provoke MSCs and ASCs to respectively secrete a wide range of cytokines, growth factors, and bioactive molecules with neurotrophic, antiapoptotic, immunomodulatory, angiogenic, re-epithelization, anti-scar, and paracrine effects, which is one of the primary mechanisms related to their potential to repair damaged tissue and regenerate [47,48,49].

MSCs also have a paracrine function thanks to their ability to secrete extracellular vesicles (EVs) that include exosomes, microvesicles, and apoptotic bodies, whose composition depends on the tissue of origin. The exosomes secreted by ASCs have a diameter of 30–100 nm and are reported to promote vascularization; however, their transplantation could be used for clinical applications in regenerative medicine [50]. Physiologically, they play an essential role in regulating biological functions, homeostasis, and the body’s immune response. The activity of microvesicles is comparable to that of MSCs [51]. Their ECVs are responsible for tissue repair even at higher magnitudes [52]. As a paracrine product of stem cells, exosomes have the same functions and are rich in proteins, mRNA, miRNA, and other substances [53]. However, there are also some specific substances that come from cells isolated from adipose tissue. Through the mass spectrometry analysis of the ASC secretome profile in humans, 342 proteins in normoxic condition were identified to be functionally related to angiogenesis and vasculature development, extracellular matrix (ECM) formation, cell adhesion/migration, cell survival/death, and immune regulation [54]. An analysis of ASC secretome composition revealed various trophic growth factors, such as vascular endothelial growth factor (VEGF), hepatocyte growth factor (HGF), insulin-like growth factor (IGF) −1, β-nerve growth factor (NGF), stromal cell-derived factor (SDF) −1α, and exosomes, which are functional in cardiovascular disease therapy [35], platelet-derived growth factor (PDGF), basic fibroblast growth factor (bFGF) [55,56], cytokines, RNAs, and lipid mediators [57].

Since adipose tissue is recognized as a major endocrine organ, the type and quantities of its products are highly dependent on the health status and individual deviations of the subjects in vivo and on microenvironment conditions in vitro. Simultaneously, as a metabolically active tissue, the secretome expression profile of ASCs varies profoundly as well. In this respect, a considerable lack of findings related to the products of equine ASCs exists, and future research is necessary to elucidate the best purpose and preconditions for using ASCs as cell-free therapy in equine regenerative medicine.

***Immunomodulatory effect of ASCs***: MSCs influence the immune T and B cellular response [58] by enhancing/exerting immunoregulatory effects on acquired and innate immune cells, such as T and B lymphocytes, dendritic cells, natural killer cells, and monocyte [58,59]. They directly suppress the activation and proliferation of immune cells [60] and also limit the synthesis of immunoglobulins, such as IgM, IgG, and IgA secreted by activated B cells, preventing their further differentiation into plasmatic cells and their ability to migrate [61]. Activated equine MSCs derived from bone marrow, adipose tissue, umbilical cord blood, and umbilical cord tissue secrete high concentrations of mediators that are similar to those of MSCs from rodents and humans in their immunomodulatory profiles [62]. The application of ASCs in both pathological and healthy equine endometrial tissues has shown some opposite effects on the regulation of inflammatory processes in the endometrium by changing the expression levels of IL1B, IL10, TNFA, IL1RN, IL6, and IL8 [63].

Aside from producing bioactive substances, MSCs migrate far from the application area and regenerate damaged tissue. Despite the effects on the immune–inflammatory response, ASCs influence regenerative processes by modulating the extracellular matrix structure. A specific family of proteins regulates this process: matrix metalloproteinases (MMPs) and their inhibitors (TIMPs), which together cause the degradation of the protein components of the extracellular matrix and, thus, can modulate the stem cells’ homing [64]. This feature of MSCs is used in trials for mares’ endometriosis treatment with allogeneic equine ASCs, where differences in the biological response are observed. They concern not only the above-mentioned pro- and anti-inflammatory factors, but also changes in the expression levels of MMP2 and TIMP2 (decreased) and MMP9 (increased) [63]. Therefore, the behavior of ASCs and the composition of secreted products will depend on the condition of the treated tissue and could provoke unexpected consequences and adverse side effects. Extracellular conditions and nutritional factors could also influence the ability of ASCs in extracellular matrix remodeling. In vitro studies in rabbits, for example, have revealed that some anti-inflammatory dietary additives, such as PUFAs (polyunsaturated fatty acids), DHA (docosahexaenoic acid), and EPA (eicosapentaenoic acid), which are PPAR-γ ligands, seem to influence the transcriptional profile of MMPs differently in subcutaneous and visceral ASCs in vitro [65,66]. In this aspect, it is necessary to balance between pro-and anti-inflammatory, lytic, and fibrotic environments [63] and estimate all factors that could potentiate the regeneration and healing processes of an injured tissue. The co-administration of nutritional anti-inflammatory factors together with cell-based and non-cell-based therapy could promote clinical efficacy.

***Factors influencing ASCs productivity and multipotency***: The development of the regenerative potential of ASCs depends on many factors. In that sense, if even one of the requirements postulated by ISCT regarding the ability of ASCs to attach to the surface of the vessel and the multipotent potential for differentiation and expression of specific surface antigens is not fulfilled, the stemness features of the cells will be questionable. Most of the induction mixtures have been adopted from human MSC differentiation protocols, but there is spice-specific responsiveness in mammals, and the concentrations and combinations of the main inducers should be reconsidered and optimized accordingly. In horses, adipogenesis as part of the tri-lineage differentiation program is a challenge. The main inductors are insulin, IBMX, dexamethasone, and indomethacin, where the commonly reported concentrations of the latter range between 0.2–0.1 mM [16,67,68]. When applied in those concentrations to equine ASCs, indomethacin causes a high level of cytotoxic effects, accompanied by a massive cellular detachment. For the successful performance of tri-lineage differentiation, the dosage of indomethacin as an adipogenic inductor should be revised to 0.05 mM [69], which in proper combinations with other inductors seems to be sufficient to preserve cellular vitality and enchase adipogenic differentiation capability in equine ASCs.

The next main component of culturing media is the serum. Currently, up-to-date testing of the consequences of the different serum types on MSC functionality is still unclear [70], and is even less so for equine ASCs. Bovine serum is mostly used in cell culture protocols, but horse serum could also be analyzed for growth factors and hormones [71]. On the one hand, FBS deprivation lowers metabolic and proliferative activity at the transcriptomic level in ASCs. However, its surplus could cause the clonal expansion of cells that have lost their ability to differentiate and do not respond to environmental inhibition [32]. FBS could also pose the hidden risk of zoonotic transmission and xeno-immunization to the recipients in clinical applications [72]. Since FBS (fetal bovine serum) could significantly alter the MSC phenotype, rendering these cells immunogenic, the bovine-derived exogenous proteins expressed on the MSC’s cellular surface may be recognized by the host immune system as non-self and thus would be rejected [73]. The culture conditions could also influence the marker expression of the MSCs, which can change the phenotype of the cells and lead to contradictory reports on marker expression [74]. It has been reported that the removal of FBS from both canine and equine MSC culture systems alters their immunomodulatory properties, and more studies are necessary before the transition to FBS-free culture conditions is effectuated [75].

In contrast, the comparison between the immunomodulatory and the antibacterial properties of equine bone marrow MSCs cultured in FBS or autologous or allogeneic equine serum has established that cells in FBS are more functionally active than those in equine serum [70]. Recently, platelet lysate has been proposed for culturing equine bone-marrow-derived MSCs as an alternative supplement to serum-free media to escape the negative consequences of FBS [73]. The influence of serum conditions on equine ASC phenotype and functionality is yet to be fully evaluated.

The origin tissue of the isolated cells can significantly affect their physiological properties, and ASCs from different sources have displayed distinct characteristics [76]. In rabbit ASCs, directly seeded cells from subcutaneous fat depots have shown a more vital ability to differentiate into adipocytes than those from visceral fat depots and their corresponding supernatants [77]. When comparing ASCs in mice and humans, significant differences in the surface markers, such as a predominant expression of CD10 in subcutaneous tissue and that of CD200 in visceral adipose tissue depots, have been established [76].

## 2. Discussion

***Clinical application of ASCs in equine disorders***

In contrast to humans, regulatory agencies do not control the clinical application of ASCs in veterinary patients and horses. The relevant preclinical studies are pure [78], and the protocols are not unified. Although the European Medicines Agency’s (EMA) Committee for Medicinal Products for Veterinary Use (CVMP) has suggested some fundamental principles for stem-cell-based treatments for animals, the unified law governing stem cell therapy usage in veterinary medicine is still missing, and each member of the European Union regulates this area autonomously [79].

Factors such as senescence, genomic stability, differentiation potential, microbiological contamination, the donors’ age, tumorigenicity [80,81], etc., should be considered when it comes to cellular therapies’ standardization and quality control [82]. Another factor of importance is the amount of the applied cellular mass. In general, it varies between 10–30 × 10^6^ and depends on the clinical condition, the disease’s specificity, the size of the lesion, and the application type (if they are applied subcutaneously, intravenously or intra-articular, for example) [3,79,83]. For example, in horses, the recommended dosage for intra-articular application for osteoarthritis is 20 × 10^6^ MSCs [84].

There are two main directions for the outcome of clinical applications: cellular-based and non-cellular therapy. In some diseases, the positive effect of ASC treatment is categorically proven.

A considerable potential has been noted for the spontaneous migration of equine ASCs toward injury sites, which is accompanied by the up-regulation of a critical musculoskeletal progenitor marker, which may be helpful in regeneration therapies for ***musculoskeletal disorders*** such as tendon and ligament injuries or osteoarthritis, which are common in horses [85,86]. In athletic horses, the flexor tendons often work close to the rupture limit, especially the superficial flexors, and under intense pressure, these degenerative injuries may lead to frequent ruptures of the tendon fibers, which can be the end of a sporting career [87,88]. Scar formation usually follows the initial inflammatory reaction that occurs at the onset of the injury. The application of allogeneic ASCs in horses leads to a lack of local inflammatory response [89], which supports the healing process. The damaged tissue is characterized by atypical mineralization, which can result in rupture upon overload due to an increased expression of type III collagen. In comparison to collagen I, which is predominant in a healthy tendon, it possesses less strength, elasticity, and resilience [90,91]. In these cases, MSC administration aims to restore standard collagen fibers and regular tendon activity, with minimal risk of recurrence [92,93]. After the combined application of ASCs with PRP (platelet-rich plasma) to the lesion of the superficial flexor, and after the completion of the rehabilitation program, a reduction of the defect, a better organization of collagen fibers, increased blood flow, and an up-to-90% recovery of treated horses have been observed [94,95,96].

Another problem in athletic horses is osteoarthritis, evidenced by lameness related to degenerative joint alteration, which usually causes complete exclusion from endurance exercising [97]. In a comparative study of the effect of the intra-articular administration of ASCs against steroid treatment in this type of injury, no inflammatory process was observed at the end of the experimental period in both group, but the improvement was noted only in the horses treated with ASCs [98].

***Wound healing*** is another attractive aspect of ASCs’ clinical application in equines, but their potential in horses are still poorly explored. In humans, an ASC exosomal concentration of 50 μg/mL promotes collagen III and I expression, suggesting that exosomes may promote wound repair by optimizing fibroblasts and, in that way, accelerate cutaneous wound healing [99]. In rabbits, the combined treatment of ASCs and plasma rich in growth factors (PRGF) could potentiate significantly and hasten the epithelialization rates and the healing process in cutaneous wounds [100]. To date, it is clear that in athletic horses, the amount of body fat is low, but even quantities from this source can yield a sufficient number of MSCs from adipose tissue; together with the above-mentioned potential of equine ASCs for spontaneous migration toward the injury sites, they might provide a benefit during wound healing by transplanted cells [85].

***Equine metabolic syndrome*** (EMS) is characterized by adiposity, insulin dysregulation, and an increased risk of laminitis. Increased levels of specific liver enzymes in the peripheral blood are typical findings in horses diagnosed with EMS. However, new potential treatment options are available, such as the transplantation of autologous ASCs [101]. In addition, in rabbit visceral ASCs in vitro, the activation of additional lipolysis pathways has been observed compared to a subcutaneous group, where EPA up-regulates the mRNA expression of lipolysis-associated genes to a greater extent than DHA [102]. Regarding the PUFAs, it has been reported that combined EPA-DHA treatment negatively affects leptin and obesity-related membrane-type MT1-MMP (MMP-14) mRNA expression in rabbit subcutaneous ASCs in vitro [66]. As an anti-inflammatory agent and as they functionally correlate with the modulators of ECM, PUFAs could play a supportive role when targeting the benefits of ASCs’ clinical application in metabolic syndrome and related disorders.

Unfortunately, the results of ASCs’ clinical application could be disappointing in some cases. By acting in a paracrine manner, ASCs accelerate tumor growth in co-cultures with cancer cells and stimulate the secretion of interleukin-6 in ASCs, which in turn causes the cancer cells to enhance their malignant properties in a paracrine manner [27,103]. A lack of positive outcomes have been observed in mare ***endometriosis***, which has resulted from chronic inflammatory damage where glandular fibrosis takes advantage of the intact tissue. The researchers reported that the application of ASCs in that case changed some of the pro-and anti-inflammatory substances, such as some interleukins, MMPs, and their TIMPs related to the extracellular matrix components, but led to no apparent clinical effects [63].

ASCs are mainly used with proven positive clinical effects in musculoskeletal disorders such as tendons and ligament injuries and in joint diseases not only in horses, but also in dogs [104]. In contrast to horses, where the ASCs have significant healing potential in aforementioned disorders, ASCs have also been successfully applied in orodental diseases in cats [105]; digestive tract diseases in dogs and cats [106,107]; and liver [108] and neuromuscular diseases such as chronic spinal cord injury [109] and keratoconjunctivitis [110] in dogs.

## 3. Conclusions

Subcutaneous adipose tissue is an easily accessible and potent source of ASCs for regenerative purposes in horses. Due to the higher yield and physiological features of stem cells related to the healing of malfunctioned tissues, their clinical application could benefit and amplify the effects of traditional treatments. It is a big challenge in veterinary medicine to classify isolated cells as stem cells due to the wide variety and specificity among animals. The adaptation, optimization, and standardization of equine-specific protocols for cell isolation and culture conditions should focus on revealing the real regenerative potential of their ASCs. The future direction of the exploration of ASCs as allogeneic transplants in veterinary medicine and as a supportive therapy should be towards the transition from cell-based to cell-free therapy. More profound studies are necessary to apply those innovative approaches to the treatment of traumatic disorders in racing horses.

## Data Availability

Not applicable.
